# Structural connectivity and subcellular changes after antidepressant doses of ketamine and Ro 25-6981 in the rat: an MRI and immuno-labeling study

**DOI:** 10.1007/s00429-021-02354-0

**Published:** 2021-08-07

**Authors:** Raquel Pascual-Antón, Arantxa Blasco-Serra, Emma Muñoz-Moreno, Fuencisla Pilar-Cuéllar, Emilio Garro-Martínez, Eva Florensa-Zanuy, Xavier López-Gil, Víctor M. Campa, Guadalupe Soria, Albert Adell

**Affiliations:** 1 Institute of Biomedicine and Biotechnology of Cantabria, IBBTEC (CSIC, University of Cantabria), Calle Albert Einstein 22 (PCTCAN), Santander, Spain; 2 GESADA Laboratory, Department of Human Anatomy and Embryology, University of Valencia, Valencia, Spain; 3 Experimental MRI 7T Unit, August Pi i Sunyer Biomedical Research Institute (IDIBAPS), Barcelona, Spain; 4 Biomedical Research Networking Center for Mental Health (CIBERSAM), Santander, Spain; 5 Department of Physiology and Pharmacology, School of Medicine, University of Cantabria, Santander, Spain; 6 Biomedical Research Networking Center in Bioengineering, Biomaterials and Nanomedicine (CIBER-BBN), Barcelona, Spain

**Keywords:** Neuroimaging, Dorsal raphe nucleus, Infralimbic cortex, Fast-acting antidepressant, Neurofilament, Myelinization

## Abstract

Ketamine has rapid and robust antidepressant effects. However, unwanted psychotomimetic effects limit its widespread use. Hence, several studies examined whether GluN2B-subunit selective NMDA antagonists would exhibit a better therapeutic profile. Although preclinical work has revealed some of the mechanisms of action of ketamine at cellular and molecular levels, the impact on brain circuitry is poorly understood. Several neuroimaging studies have examined the functional changes in the brain induced by acute administration of ketamine and Ro 25-6981 (a GluN2B-subunit selective antagonist), but the changes in the microstructure of gray and white matter have received less attention. Here, the effects of ketamine and Ro 25-6981 on gray and white matter integrity in male Sprague–Dawley rats were determined using diffusion-weighted magnetic resonance imaging (DWI). In addition, DWI-based structural brain networks were estimated and connectivity metrics were computed at the regional level. Immunohistochemical analyses were also performed to determine whether changes in myelin basic protein (MBP) and neurofilament heavy-chain protein (NF200) may underlie connectivity changes. In general, ketamine and Ro 25-6981 showed some opposite structural alterations, but both compounds coincided only in increasing the fractional anisotropy in infralimbic prefrontal cortex and dorsal raphe nucleus. These changes were associated with increments of NF200 in deep layers of the infralimbic cortex (together with increased MBP) and the dorsal raphe nucleus. Our results suggest that the synthesis of NF200 and MBP may contribute to the formation of new dendritic spines and myelination, respectively. We also suggest that the increase of fractional anisotropy of the infralimbic and dorsal raphe nucleus areas could represent a biomarker of a rapid antidepressant response.

**Supplementary Information:**

The online version contains supplementary material available at 10.1007/s00429-021-02354-0.

## Introduction

Depression is the most prevalent of psychiatric diseases with a high medical and societal burden. Current antidepressant treatments fail to achieve full response in approximately 40% of patients. Therefore, there is a crucial need to develop new and rapid therapies. Ketamine exhibits robust and rapid antidepressant effects. However, its early psychotomimetic effects precede antidepressant action. For this reason, several studies examined whether GluN2B-subunit selective NMDA receptor antagonists would exhibit a better therapeutic profile. Although preclinical work has uncovered some of the mechanisms of action of ketamine at cellular and molecular level, the way the drug impact on brain circuitry is poorly understood.

Initial clinical trials showed some efficacy of GluN2B subunit antagonists (Preskorn et al. 2008; Ibrahim et al. 2012) although none of these novel investigational drugs exhibited an improved efficacy in comparison to ketamine. On the other hand, preclinical experiments in rodents demonstrated that both ketamine and the GluN2B subunit antagonist, Ro 25-6981, had rapid-acting antidepressant-like effects (Li et al. 2010; Jiménez-Sánchez et al. 2014; Kiselycznyk et al. 2015). Several neuroimaging studies have addressed the issue of functional changes in the brain induced by acute administration of ketamine and Ro 25-6981. For instance, results from recent positron-emission tomography (PET) studies in humans reported that a single intravenous bolus infusion of ketamine increased glucose consumption in dorsal anterior cingulate and prefrontal cortices and hippocampus, changes that correlated with amelioration of depressive symptoms (Carlson et al. 2013; Lally et al. 2015; Li et al. 2016). In rats, both ketamine and Ro 25-6981 induced increases in blood oxygen level-dependent (BOLD) functional connectivity of brain regions controlling mood and reward processes (Gass et al. 2014, 2018). Nevertheless, although preclinical and clinical work has revealed important functional changes after acute ketamine administration (Ionescu et al. 2018), less attention has been paid to microstructural changes as a result of NMDA receptor blockade despite the fact that alterations in cortico-subcortical circuitry in depression are well established (Price and Drevets 2012). Furthermore, few studies have revealed white matter (WM) abnormalities in depression that correlated with the severity of the pathology (Cole et al. 2012; Vasavada et al. 2016), and a more recent study has evidenced a disconnection between the prefrontal cortex and the rest of the brain in depressive subjects (Abdallah et al. 2017). Overall, preclinical and clinical work has revealed important functional changes after acute ketamine administration and a vast majority of structural neuroimaging studies in depression have focused on the large WM tracts. However, subtle microstructural changes in cortical and subcortical regions may also lead to disturbances on brain connectivity in depression.

To address this question, here we examined the effects of ketamine and Ro 25-6981 on structural integrity using diffusion-weighted magnetic resonance imaging (DWI) using male Sprague–Dawley rats. DWI measures the diffusion of water molecules in brain tissue, which is related to the underlying structure. A diffusion tensor model was fitted to the DWI acquisition to obtain the diffusion tensor image (DTI) and estimate diffusion metrics that included fractional anisotropy (FA), which quantifies diffusion anisotropy; mean diffusivity (MD), which measures the amount of diffusion regardless its direction; axial diffusivity (AD), which measures the diffusion in the predominant direction; and radial diffusivity (RD), which measures the diffusion perpendicular to the main direction (Le Bihan et al. 2001; O’Donnell and Westin 2011; Soares et al. 2013). The use of these four different measures is crucial to revealing the characteristics of tissue microstructure. In addition, DWI-based structural brain networks were estimated and connectivity metrics were computed at regional (nodal) level. Further, we have used pre-drug baseline values in the same animals for studying longitudinal changes. Because diffusion metrics does not provide information about the underlying changes in the microstructure of brain tissue, we have performed immunohistochemical (IHC) analyses to determine whether changes in the values of these metrics could be attributed to changes in myelin basic protein (MBP) and/or neurofilament heavy-chain (200 kDa) protein (NF200), two biomarkers of axonal myelination and cytoskeleton microstructure, respectively.

## Methods

### Animals and drugs

Adult male Sprague–Dawley rats (Envigo) weighing 300–350 g (9–11-week-old) were group housed on a 12 h light/dark cycle (lights on 08:00 h) with food and water freely available. All procedures were done in accordance with national (RD 53/2013) and European legislation (Directive 2010/63/EU, on the Protection of Animals Used for Scientific Purposes, 22 September 2010), and were approved by the Institutional Animal Care and Use Committees. Ketamine hydrochloride (Ketolar^®^) was purchased from Pfizer and diluted to 25 mg/ml in saline for intraperitoneal (i.p.) injection. Ro 25-6981 maleate was purchased from Tocris Biosciences (Abingdon, UK), diluted to 10 mg/ml in 50% dimethyl sulfoxide (DMSO)/water and injected i.p. Both ketamine and Ro 25-6981 were injected at a volume of 1 ml/kg. Although the dose of 10 mg/kg of ketamine already showed antidepressant-like effects in rats (Li et al. 2010; López-Gil et al. 2019), we chose the dose of 25 mg/kg (i.p.) because it produced a more sustained elevation of extracellular glutamate levels in the mPFC (Moghaddam et al. 1997; López-Gil et al. 2019). The dose of Ro 25-6981 (10 mg/kg, i.p.) was chosen from previous studies that showed antidepressant-like effects in behavioral tests (Li et al. 2010; Jiménez-Sánchez et al. 2014).

### Magnetic resonance imaging (MRI)

Magnetic resonance image (MRI) experiments were conducted on a 7.0 Tesla BioSpec 70/30 horizontal animal scanner (Bruker BioSpin, Ettlingen, Germany), equipped with an actively shielded gradient system (400 mT/m, 12 cm inner diameter). The receiver coil was a 4-channel phased-array surface coil for the rat brain.

Animals were anesthetized in a methacrylate chamber with an induction dose of 4% isoflurane in a mixture of 30% O_2_ and 70% N_2_O. Afterwards, they were placed in supine position in a Plexiglas holder with a nose cone for administering anesthetic gases and fixed using tooth and ear bars and adhesive tape. Eyes were protected from dryness with Siccafluid 2.5 mg/g ophthalmologic fluid. Once placed in the holder, anesthesia (1.5% isoflurane in a mixture of 30% O_2_ and 70% N_2_O) was maintained during all the scan protocol. Three different MRI scans were performed. The first scan was performed 7 days before drug administration and was considered as pre-drug baseline. The second scan was performed 24 h after drug administration and the last scan was performed 7 days after drug administration. The two latter scans were conducted at time points in which previous clinical studies have demonstrated robust antidepressant effects of ketamine (Zarate et al. 2006). 3D-localizer scans were used to ensure accurate positioning of the head in the magnetic isocenter. Anatomical T2-weighted images were acquired with rapid acquisition with relaxation enhancement (RARE) sequence with effective echo time TE = 33 ms, repetition time TR = 3610.784 ms, RARE factor = 8, pixel size: 0.137 × 0.137 mm^2^, 34 slices, slice thickness = 0.8 mm, field of view (FoV = 35 × 35 × 27.2 mm^3^). DWI images were obtained using a spin echo-planar imaging (EPI) sequence (TE = 24.86 ms, TR = 15,000 ms, 4 segments, 60 gradient directions with *b* value = 1000 s/mm^2^ and 5 volumes with *b* value = 0 s/mm^2^; matrix size = 72 × 72 pixels; FoV = 22.23 × 22.23 mm^2^, which resulted in a real isotropic acquisition of 0.31 × 0.31 × 0.31 mm^3^ voxel size. DWI was processed using DIPY software, which include Eddy current correction, de-noising and fitting of the diffusion tensor model to the data (Garyfallidis et al. 2014).

The diffusion tensor metrics (FA, RD, MD, AD) were then computed and averaged from the value of all containing voxels within each of the 13 regions of interest (ROIs). These ROIs were manually delineated on each subject brain to obtain accurate segmentation of the areas. A rat brain atlas was used as reference (Paxinos and Watson 2007) and the drawing of the ROIs were performed with the ITK-SNAP software (Yushkevich et al. 2006). The brain areas examined were dorsal raphe nucleus (DRN), infralimbic (IL), prelimbic (PrL), anterior cingulate (ACg), and orbitofrontal (OFC) cortices as well as dorsal (d) and ventral (v) hippocampus (HPC), amygdala (Amy), nucleus accumbens (NAcc), thalamus (THL), lateral striatum (lSTR), medial striatum (mSTR) and corpus callosum (CC). We have divided the striatum into lateral and medial subareas because it has been suggested that changes in the network topology of these subareas occur differently in MDD, possibly due to specific intrinsic connectivity (Meng et al. 2014).

### Brain connectivity analysis

The structural connectivity between regions of interest was estimated to build connectome matrices and graph theory was applied to characterize network organization (Sporns 2012). To build the connectome, ROIs previously delineated on the DWI volumes were considered as network nodes. Deterministic tractography based on a constrained spherical deconvolution model was performed to estimate the fiber tracts in the whole brain. Voxels with FA > 0.1 were considered as seed points and the same value was considered as stop criterion. Processing was performed using DIPY software (Garyfallidis et al. 2014). Two regions A and B were considered to be connected if at least one streamline started in A and ended in B (Hagmann et al. 2008). To quantify the strength (or weight) of the connection, fiber density (FD) was considered. It is defined as the number of streamlines connecting two regions divided by the region volumes and the streamline length (Batalle et al. 2014). Thus, the FD-weighted connectome was built, where each element represents the FD of the connection between a pair of regions. In addition, a normalized FD connectome (FD-n) was also considered. In this case, the FD of each individual connection is normalized by the total strength of the FD-weighted connectome, that is, by the sum of FD of all the connections in the network. Hence, the FD-n allows to assess the brain organization independently of the overall network strength (Batalle et al. 2014).

Regional connectivity was evaluated by graph theory metrics, namely strength, efficiency and clustering coefficient. Nodal strength measures the total weight of the connections of one region; nodal efficiency quantifies the ability to transfer information in the subnetwork associated to that region, and it is inversely related with the shortest path length between each pair of regions in the subnetwork. Finally, clustering coefficient measures the number of node neighbors that are also neighbors to each other (Sporns 2012). Regional network metrics were computed for each acquisition (subject and time point). FD-n nodal efficiency and clustering coefficient were used in the present work to examine changes in brain connectivity after ketamine and Ro 25-6981.

### Histology and immunohistochemistry

MBP and NF200 immunoreactivity was performed in sections of the three subregions of the mPFC (namely ACg, PrL and IL) and DRN of rats killed 24 h after injection of ketamine (25 mg/kg), Ro 25-6981 (10 mg/kg) or vehicle (50% DMSO). This time point was chosen because we were interested in the early effects of both drugs and, also, because it was when ketamine produced the maximal effects in the antidepressant activity (Zarate et al. 2006) and the formation of new synaptic boutons in pyramidal cells of the medial prefrontal cortex (mPFC) (Li et al. 2010). 24 hours after the injection of ketamine, Ro 25-6981 or 50% DMSO, the animals were anesthetized with sodium pentobarbital and transcardially perfused with 0.9% NaCl for 15 min followed by 4% paraformaldehyde (PFA) in phosphate buffered saline (PBS) for 15 min. Then, the brains were removed from the skull, immersed in 4% PFA in PBS for 4 h and, finally, tissue was cryoprotected in 30% (wt/vol) sucrose in PBS for 48 h. Then, brains were frozen on dry ice and stored at – 20 °C until cutting. Coronal cryosections (40 µm, 1-in-6 series) were collected into cryoprotectant medium (125 ml ethylene glycol, 125 ml glycerol, 50 ml PBS 10 × and 200 ml of distilled water) and stored at – 20 °C until processing.

Immunohistochemistry was performed on free-floating sections. First, sections were washed with PBS (3 times, 5 min each) and non-specific binding was blocked in incubation solution [0.3% Triton X-100, 2% bovine serum albumin (BSA) in PBS] containing 3% normal donkey serum (NDS) for 1 h at room temperature (RT). Then, sections were incubated with mouse anti-myelin basic protein (MAB381, a.a. 119–131, clone 2; 1:50) and rabbit anti-neurofilament heavy chain (N4142, NF200 antibody; 1:400)—both purchased from Sigma-Aldrich (St. Louis, MO)—diluted in incubation solution overnight at 4 °C, washed with PBS (3 times, 5 min each) and incubated for 2 h at RT with Alexa Fluor^®^ 488 donkey anti-mouse IgG (1:200; Invitrogen), Alexa Fluor^®^ 568 donkey anti-rabbit IgG (1:200; Invitrogen) in incubation solution. Finally, sections were counterstained with 4’,6-diamidino-2-phenylindole (DAPI, 1:1000), washed with PBS (3 times, 5 min each) and mounted with Vectashield (Vector Laboratories, Burlingame, CA).

Following immunofluorescent staining, high-magnification confocal images showing neurofilament and myelin structure were acquired on a SP5 laser-scan microscope (Leica) with a 40/1.25 NA objective by averaging three scans (frame averaging) using LAS AF acquisition software. Cells were excited sequentially with 405 nm, 488 nm and 532 nm laser lines and emission captured between 415 and 484 nm (DAPI), 500–550 nm (Alexa 488) and 605–650 nm (Alexa 568) avoiding detector saturation. After the DRN and IL were identified at low magnification, 11 planes (5.04 µm thick) and 1024 × 1024 pixels (0.076 µm/pixel) Z-stacks were acquired. Maximum intensity projection of the Z-stacks is presented after digital adjustment of brightness and contrast to maximize signal. Quantification of the signals was performed with Fiji software (Schindelin et al. 2012). In all cases, exposure time, sensor gain, and image contrast adjustment were the same for control and experimental samples. Fluorochromes and colors are as indicated in the corresponding figure legends.

### Statistics

Differences in diffusion scalars (FA, RD, MD, and AD), nodal efficiency and clustering coefficient were assessed by repeated measures analysis of variance (ANOVA) with treatment, time, region and hemisphere as main factors, followed by Duncan’s tests corrected for multiple comparisons. Optical density values in IHC analyses were assessed by one-way ANOVA followed by Duncan’s tests corrected for multiple comparisons. Statistical significance was set at *p* < 0.05. Statistical analyses were performed with Statistica Version 10 statistic software package (Stat Soft, Inc., Tulsa, OK).

## Results

### Diffusion metrics

**Fig. 1:**
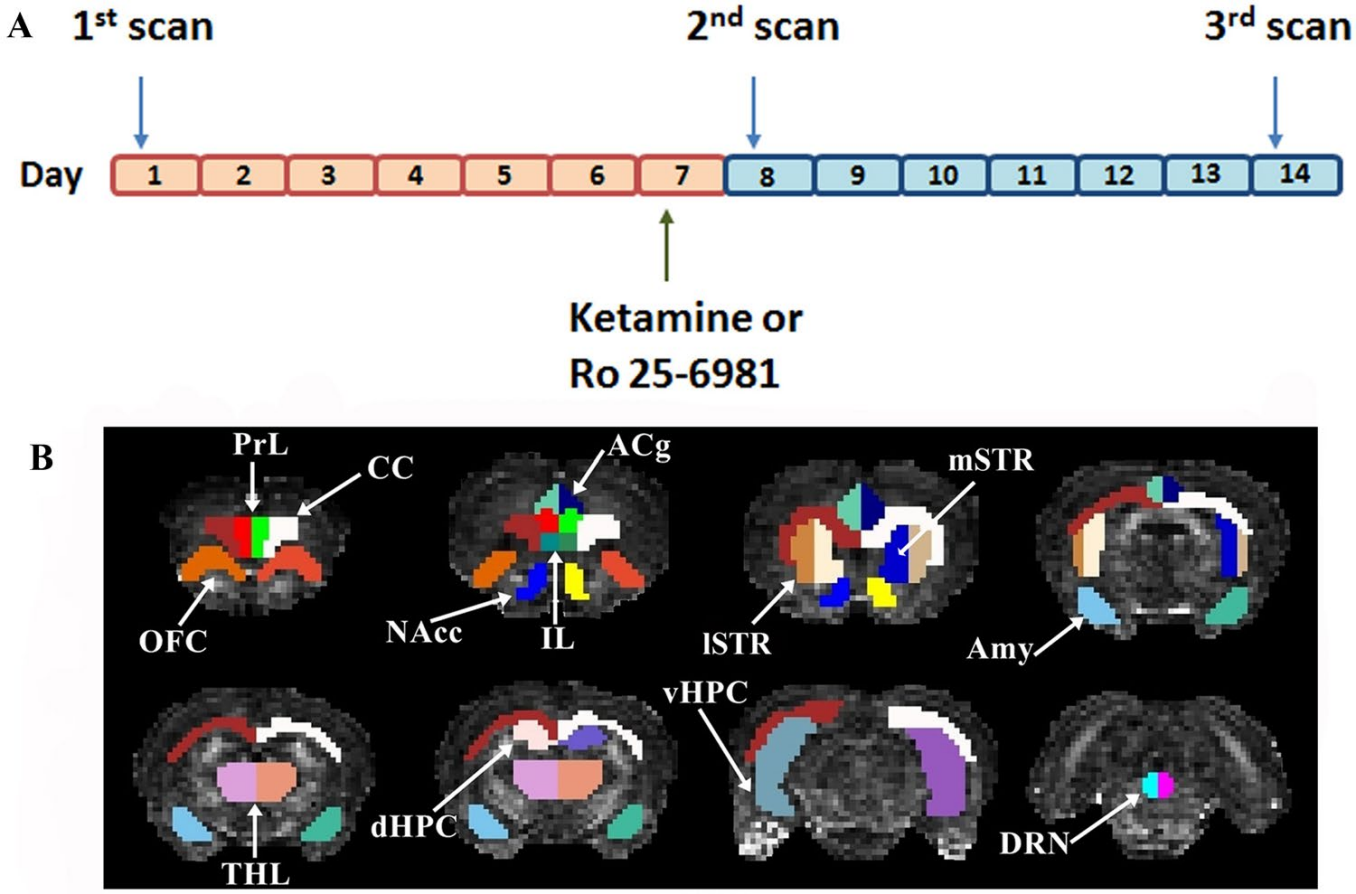
Experimental procedure for DWI analysis. **A** Timeline for DWI scans. **B** Coronal DWI images showing pseudo-colored, hand-drawn regions of interest (ROIs). Abbreviations: *PrL* prelimbic cortex; *CC* corpus callosum; *OFC* orbitofrontal cortex; *ACg* anterior cingulate cortex; *IL* infralimbic cortex; *NAcc* nucleus accumbens; *lSTR* lateral striatum; *mSTR* medial striatum; *Amy* amygdala; *THL* thalamus; *dHPC* dorsal hippocampus; *vHPC* ventral hippocampus and *DRN* dorsal raphe nucleus

The time-course of the MRI acquisitions is depicted in Fig. 1A. The 13 structures selected from each hemisphere for statistical analysis are illustrated in Fig. 1B.

**Fig. 2:**
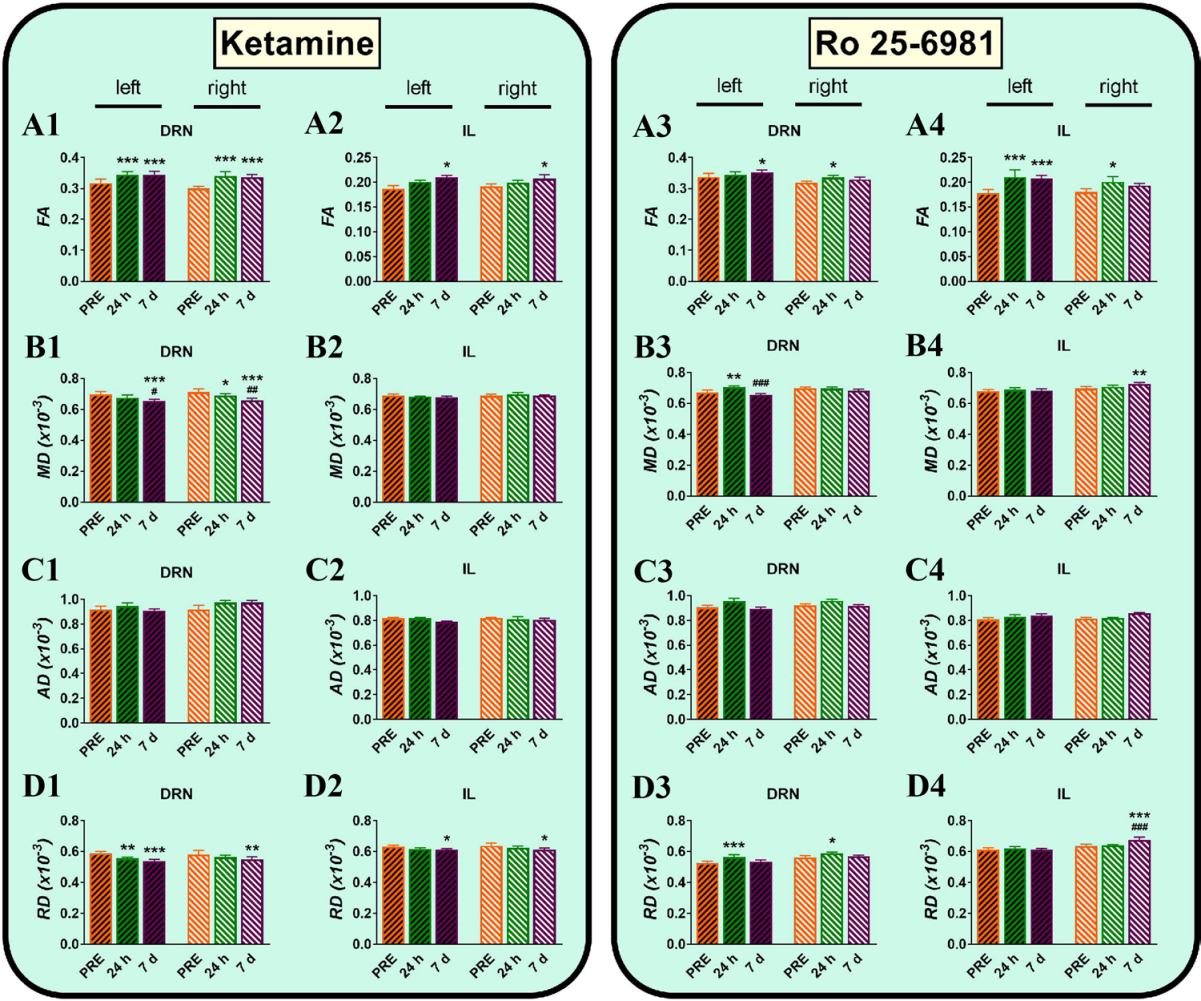
Effects of ketamine (25 mg/kg) and Ro 25-6981 (10 mg/kg) on fractional anisotropy (FA; **A1**–**A4**), mean diffusivity (MD; **B1**–**B4**), axial diffusivity (AD; **C1**–**C4**) and radial diffusivity (RD; **D1**–**D4**) in the infralimbic cortex (IL) and (**A2**, **A4**, **B2**, **B4**, **C2**, **C4**, **D2**, **D4**) the dorsal raphe nucleus (DRN) (**A1**, **A3**, **B1**, **B3**, **C1**, **C3**, **D1**, **D3**) in right and left hemispheres. The three scans were conducted 7 days before drug treatment (PRE), and 24 h and 7 days (7 days) after drug administration. Data are expressed as mean ± SEM of 5 rats. *p < 0.05, **p < 0.01, ***p < 0.001 compared to PRE group, ^#^*p* < 0.05, ^##^*p* < 0.01, ^###^*p* < 0.001 compared to 24 h group; Duncan’s multiple comparisons test following ANOVA

**Fig. 3:**
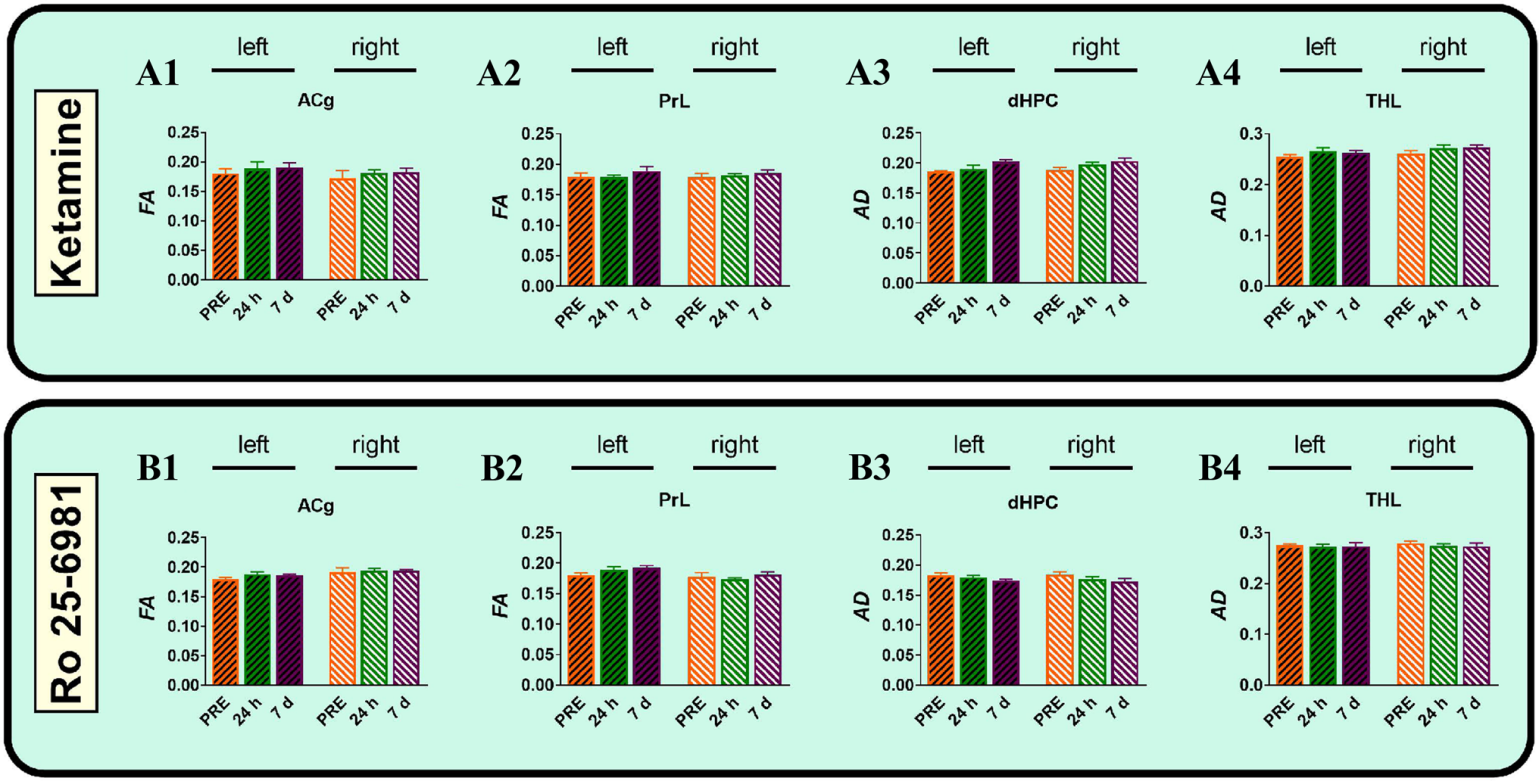
Effects of 25 mg/kg of ketamine (**A1**–**A4**) and 10 mg/kg Ro 25-6981 (**B1**–**B4**) on fractional anisotropy (FA) in the anterior cingulate cortex (ACg) (**A1**, **B1**), prelimbic cortex (PrL) (**A2**, **B2**), dorsal hippocampus (dHPC) (**A3**, **B3**), and thalamus (THL) (**A4**, **B4**) in right and left hemispheres. The three scans were conducted 7 days before drug treatment (PRE), and 24 h and 7 days after drug administration. Data are expressed as mean ± SEM of 5 rats. No significant difference was observed between drug treatments at any time point

Repeated measures ANOVA of FA values showed significant effects of region (*F*_12,208_ = 1071.8, *p* < 0.00001), and time (*F*_2,416_ = 3.3, *p* < 0.05). Significant interactions were also found for region × treatment (*F*_12,208_ = 3.3, *p* < 0.0005), region × time (*F*_24,416_ = 7.5, *p* < 0.00001), hemisphere × time (*F*_2,416_ = 3.6, *p* < 0.05), treatment × time (*F*_2,416_ = 9.8, *p* < 0.0001) and treatment × region × time (*F*_24,416_ = 2.2, *p* < 0.001). Post hoc multiple comparisons evidenced that ketamine significantly increased FA in DRN (Fig. 2, A1), IL (Fig. 2, A2), vHPC and CC in both hemispheres as well as in right Amy, NAcc and OFC (Supplementary Table S1). Ro 25–6981 also increased FA values in DRN (Fig. 2, A3) and IL (Fig. 2, A4) but, contrary to ketamine, decreased FA in Amy and OFC bilaterally (Supplementary Table S2). The changes in FA that were similar for ketamine and Ro 25–6981, i.e. DRN and IL are depicted in Fig. 2. Representative regions in which ketamine and Ro 25–6981 produced no changes in FA are represented in Fig. 3.

Repeated measures ANOVA of RD values showed significant effects of region (*F*_12,208_ = 240.2, *p* < 0.00001) and time (*F*_2,416_ = 7.4, *p* < 0.001). Significant interactions were also found for region × hemisphere (*F*_12,208_ = 2.3, *p* < 0.01), hemisphere × treatment (*F*_1,208_ = 4.7, *p* < 0.01) and treatment × time (*F*_2,416_ = 173.8, *p* < 0.00001). Post hoc multiple comparisons evidenced that ketamine significantly decreased RD values in DRN (Fig. 2, D1), IL (Fig. 2, D2), Amy, THL and CC, bilaterally. Significant decreases were also observed in the ACg, dHPC and NAcc of the right hemisphere (Supplementary Table S1). On the contrary, Ro 25–6981 increased RD values of DRN (Fig. 2, D3), ACg, vHPC, Amy, NAcc, OFC and CC in both hemispheres as well as the right IL (Fig. 2, D4 and Supplementary Table S2).

Repeated measures ANOVA of MD values showed significant effects of region (*F*_12,208_ = 138.3, *p* < 0.00001), treatment (*F*_1,208_ = 4.4, *p* < 0.05) and time (*F*_2,416_ = 14.8, *p* < 0.00001). Significant interactions were also found for region × hemisphere (*F*_12,208_ = 2.7, *p* < 0.005), region × time (*F*_24,416_ = 3.9, *p* < 0.00001) and treatment × time (*F*_2,416_ = 100.6, *p* < 0.00001). Post hoc multiple comparisons showed that ketamine significantly decreased MD in right and left DRN (Fig. 2, B1) and right THL (Supplementary Table S1). In contrast, Ro 25–6981 produced widespread, bilateral increases of MD values in ACg, Amy, NAcc and CC (Supplementary Table S2). Significant MD increases were also found in left DRN (Fig. 2, B3), right IL (Fig. 2, B4), left dHPC, right vHPC, right thalamus and right STR after Ro 25–6981 administration (Supplementary Table S2).

Finally, repeated measures ANOVA of AD values showed significant effects of region (*F*_12,208_ = 41,904.2, *p* < 0.00001), treatment (*F*_1,208_ = 41,912.5, *p* < 0.00001) and hemisphere (*F*_1,208_ = 41,903.1, *p* < 0.00001). Significant interactions were also found for region × hemisphere (*F*_12,208_ = 41,907.2, *p* < 0.00001), region × treatment (*F*_12,208_ = 41,907.8, *p* < 0.00001), hemisphere × treatment (*F*_1,208_ = 41,906.6, *p* < 0.00001) and region × hemisphere × treatment (*F*_12,208_ = 41,906.7, *p* < 0.00001). Post hoc multiple comparisons showed that only ketamine produced a significant increase of AD in the right vHPC (Supplementary Table S1) with no change in AD caused by Ro 25–6981 (Supplementary Table S2).

### Immunohistochemistry

**Fig. 4:**
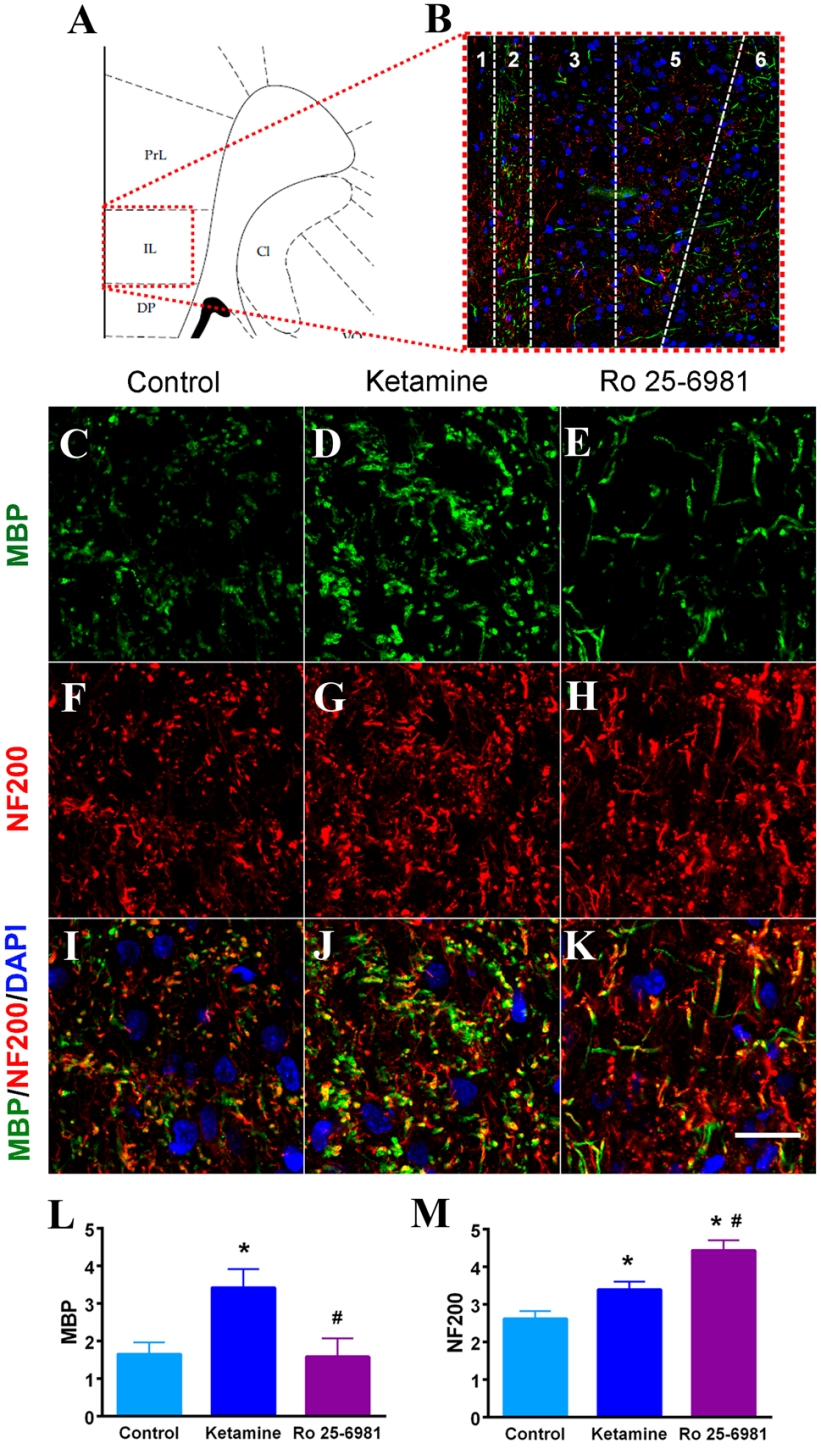
Changes in MBP and NF200 induced by ketamine and Ro 25-6981 in infralimbic (IL) cortex. **A** Graphical scheme of the prefrontal cortex area. The red dotted square indicates the area analyzed. **B** Representative image of the infralimbic cortex showing the MBP (green), NF200 (red) and cell nuclei (blue) immunolabeling. The different cortical layers are labeled as 1–6. Representative confocal images of immunolabeling for MBP (**C–E**), NF200 (**F–H**) and merged images (**I–K**), of the control (**C**, **F** and **I**), ketamine (**D**, **G** and **J**) and Ro 25-6981 (**E**, **H**, **K**) groups. Scale bar: 200 µm. Graphs show the MBP (**L**) and NF200 (**M**) analyses. Data are expressed as mean ± SEM. The statistical analysis was performed using a one-way ANOVA, followed by a Duncan’s multiple comparisons test. *p < 0.05 vs. control group, ^#^*p* < 0.05 vs. ketamine group. *n* = 6 animals per group

**Fig. 5:**
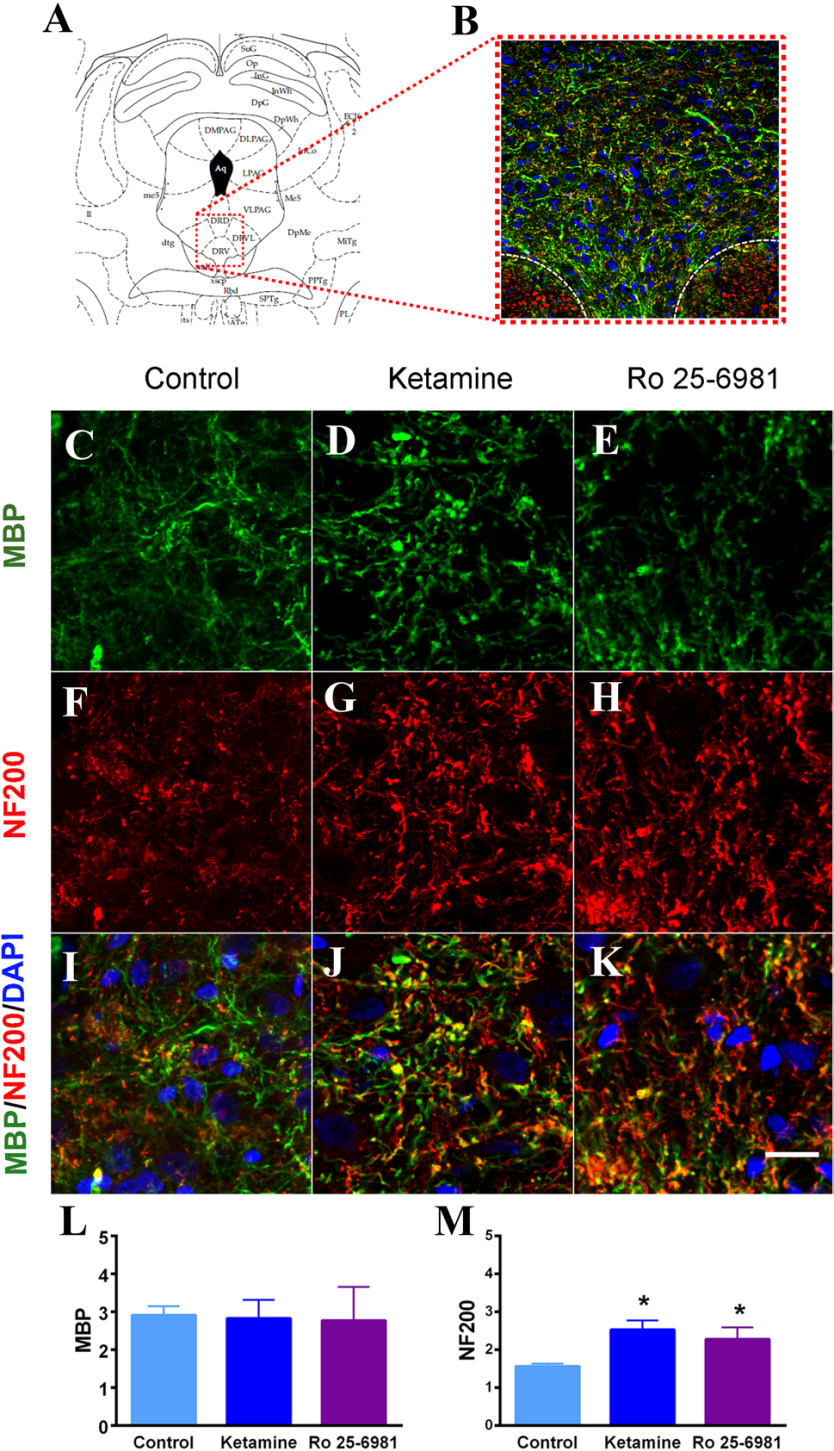
Changes in MBP and NF200 induced by ketamine and Ro 25-6981 in the dorsal raphe nucleus (DRN). **A** Graphical scheme of the DRN area. The red dotted square indicates the area analyzed. **B** Representative image of the DRN showing the MBP (green), NF200 (red) and cell nuclei (blue) immunolabeling. Representative confocal images of immunolabeling for MBP (**C–E**), NF200 (**F–H**) and merged images (**I–K**), of the control (**C**, **F** and **I**), ketamine (**D**, **G** and **J**) and Ro 25-6981 (**E**, **H**, **K**) groups. Scale bar: 200 µm. Graphs show the MBP (**L**) and NF200 (**M**) analyses. Data are expressed as mean ± SEM. The statistical analysis was performed using a one-way ANOVA, followed by a Duncan’s multiple comparisons test. ^*^p < 0.05 vs. control group. *n* = 6 animals per group

To determine the substrate(s) possibly responsible for the changes observed in DWI images after ketamine and Ro 25-6981, immunostaining for NF200 and MBP was performed in the IL (Fig. 4) and the DRN (Fig. 5), the brain areas where the effects of both drugs on FA were coincident. Also, NF200 and MBP were determined in other subareas of the mPFC, i.e. ACg and PrL (Supplementary Fig. S1 and Supplementary Fig. S2). Since only a subpopulation of pyramidal cells localized to layer 5 of the mPFC project to subcortical structures (e.g. the DRN), NF200 and MBP were measured separately in the superficial layers (1–3) (Supplementary Fig. S1) and deep layers (5 and 6) (Fig. 4 and Supplementary Fig. S2). Ketamine, but not Ro 25-6981, increased significantly MBP immuno-labeling in deep layers of IL with respect to vehicle-injected (control) animals (Fig. 4L). Both ketamine and Ro 25-6981 increased NF200 in deep layers of IL (*F*_2,11_ = 15.67, *p* < 0.001) (Fig. 4M). Nevertheless, neither ketamine nor Ro 25-6981 modified MBP and NF200 in superficial layers of IL (Supplementary Fig. S1). In the DRN, no change in MBP was observed after the administration of ketamine or Ro 25-6981 (Fig. 5L), but both ketamine and Ro 25-6981 increased NF200 in the DRN (*F*_2,10_ = 5.658, *p* < 0.03) (Fig. 5M).

### Structural connectivity analysis

**Fig. 6:**
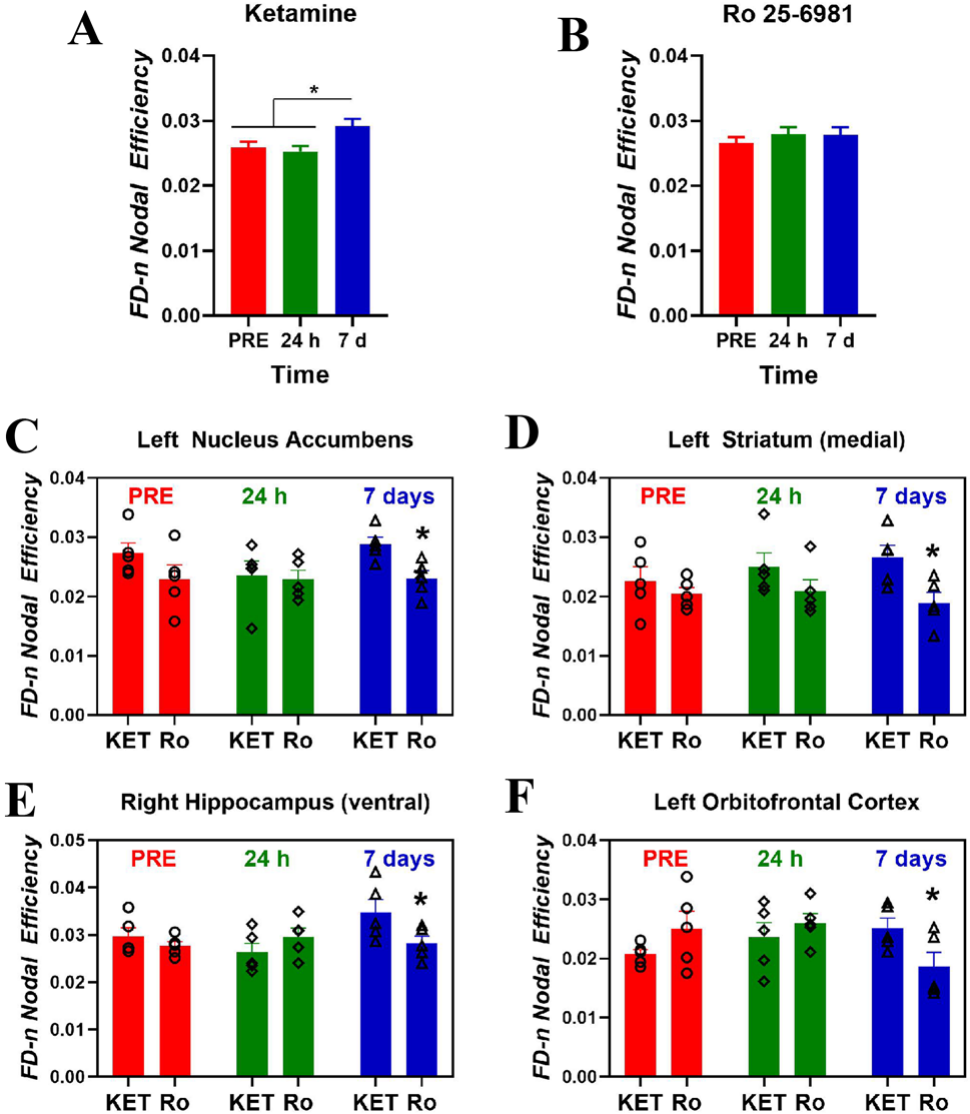
Effects of ketamine, KET (**A**) and Ro 25-6981, Ro (**B**) on nodal efficiency of the fiber density normalized (FD-n) connectome considering the thirteen brain regions as a whole. Nodal efficiencies in the left nucleus accumbens (**C**), left medial striatum (**D**), right ventral hippocampus (**E**) and left orbitofrontal cortex (**F**) showed significant differences between ketamine and Ro 25-6981 only seven days after drug treatment (*p < 0.05, Duncan’s multiple comparisons test following ANOVA). Data are expressed as mean ± SEM of five rats per group. Scans were performed 7 days before drug administration (PRE) and 24 h after and 7 days after drug administration

The analysis of regional nodal efficiency showed significant effects of region (*F*_12,208_ = 119.42, *p* < 0.00001), time *F*_2,416_ = 8.10, *p* < 0.001), and the interactions region × time (*F*_24,416_ = 1.83, *p* < 0.02) and treatment × time (*F*_2,416_ = 5.72, *p* < 0.01). When considering the 13 regions as a whole, ketamine significantly increased nodal efficiency 7 days after drug administration (*p* < 0.05, Duncan’s multiple comparison test) relative to pre-drug and 24 h values (Fig. 6A), an effect not observed after Ro 25–6981 (Fig. 6B). Post hoc Duncan’s analyses showed that, 1 week after treatment, ketamine induced greater nodal efficiency than Ro 25–6981 in the left nucleus accumbens (Fig. 6C), left medial striatum (Fig. 6D), right ventral hippocampus (Fig. 6E) and left orbitofrontal cortex (Fig. 6F).

**Fig. 7:**
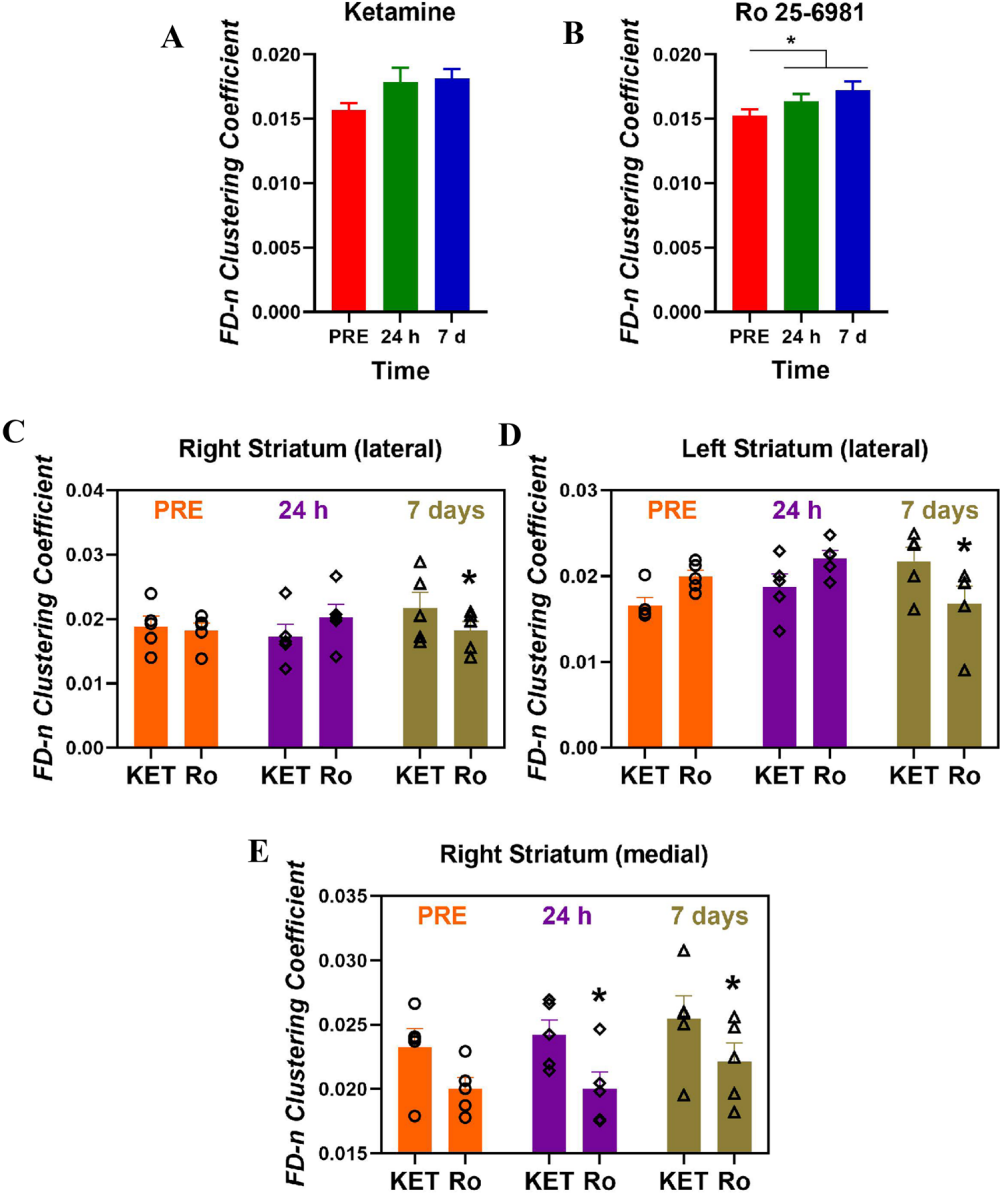
Effects of ketamine, KET (**A**) and Ro 25-6981, Ro (**B**) on clustering coefficient of the fiber density normalized (FD-n) connectome considering the thirteen brain regions as a whole. Clustering coefficients in the right lateral striatum (**C**), left lateral striatum (**D**) and right medial striatum (**E**) showed significant differences between ketamine and Ro 25-6981 only seven days after drug treatment (*p < 0.05, Duncan’s multiple comparisons test following ANOVA). Data are expressed as mean ± SEM of five rats per group. Scans were performed 7 days before drug administration (PRE) and 24 h after and 7 days after drug administration

The analysis of regional clustering coefficient showed significant effects of region (*F*_12,208_ = 47.68, *p* < 0.00001), treatment (*F*_1,208_ = 4.49, *p* < 0.05) and time (*F*_2,416_ = 8.11, *p* < 0.001), and the interactions region × treatment (*F*_12,208_ = 2.18, *p* < 0.02) and region × time (F_24,416_ = 1.61, *p* < 0.05). When considering the 13 regions as a whole, ketamine had only a marginal effect (*p* < 0.0506) on the clustering coefficient with a trend to increase after administration (Fig. 7A). In contrast, as shown in Fig. 7B, Ro 25–6981 increased the clustering coefficient both 24 h (*p* < 0.05, Duncan’s multiple comparison test) and 7 days after drug administration (*p* < 0.0005, Duncan’s multiple comparison test) relative to pre-drug values. *Post hoc* Duncan’s analyses also showed that, ketamine possessed greater clustering coefficient than Ro 25–6981 in the right (Fig. 7C) and left lateral striatum (Fig. 7D), and the right medial striatum (Fig. 7E).

## Discussion

Both ketamine and Ro 25-6981 have been reported to possess antidepressant-like actions in rodents (Maeng et al. 2008; Li et al. 2010, 2011). Although it is known that ketamine can produce hemodynamic and respiratory alterations (Forsyth et al. 2020), the changes induced in heart rate and respiratory volume peak at 2–4 min after its administration (Forsyth et al. 2020) whereas its half-life in Sprague–Dawley rats is less than 1 h (Wang et al. 2011). On the other hand, Ro 25-6981 is neuro-protectant with a short half-life (Fischer et al. 1997; Dong et al. 2017) and the absence of GluN2B subunits in the adult heart suggests a reduced probability of cardiovascular side effects (Seeber et al. 2000). Therefore, we can rule out the possibility that such physiological changes might have influenced our DTI data acquisition after drug administration. The examination of FA values together with other diffusivity parameters can allow for accurate readouts of the tissue microstructure. Thus, the configuration of increased FA/decreased RD is usually taken as a proxy of increased fiber density and/or organization (Assaf and Pasternak 2008) and/or activity-dependent myelination processes (Klawiter et al. 2011; Moore et al. 2017).

Here we demonstrate that a single intraperitoneal injection of ketamine produced elevations of FA in the DRN, IL, vHPC and CC of both hemispheres, with concomitant and more widespread reductions in RD. On the other hand, neither ketamine nor Ro 25-6981 evoked changes in AD, which suggests the absence of axonal loss (Hoogenboom et al. 2019). Ketamine also increased FA in Amy, NAcc and OFC, but only in the right hemisphere. Although it is not known the reason of this asymmetric response to ketamine, it may be due to different hemispheric subunit expression of NMDARs and/or different synaptic density (Kawakami et al. 2003; Wu et al. 2005; Shinohara et al. 2008; Capper-Loup et al. 2009). These results are also coincident with ketamine-induced increase in BOLD signal in the same areas of the rat brain (Tang et al. 2018). Unfortunately, the study by Tang and co-workers did not discriminate between both hemispheres.

Our results with naïve rats are also coherent with clinical data showing decreased FA in tracts originating from the DRN in depression (Delorenzo et al. 2013) as well as increased RD and MD in depressed patients (Cole et al. 2012), particularly in frontal lobes (Sexton et al. 2009) and also with increased FA from depressed individuals that responded to a single infusion of ketamine (Vasavada et al. 2016). Also, it has been found in humans that increased right OFC volume is positively correlated to pleasurable feelings (Rankin et al. 2004) and that an enhanced activity of the right NAcc helps to cope with external behavioral contingencies (Zhang et al. 2017), both processes being relevant to ameliorate depressive symptoms. In addition, increased FA in tracts to the right amygdala has been described in depressed people that responded to SSRI treatment (Delorenzo et al. 2013), which is also consistent with our findings. Interestingly, the increase in FA occurred first in the DRN and right NAcc (24 h after drug administration), whereas the same effect in other brain areas innervated by serotonergic neurons were observed 1 week after ketamine administration. Furthermore, the increased FA in the DRN was still present 1 week after ketamine administration, which concurs with the duration of the antidepressant effects in rats (Carreno et al. 2019) and humans (Berman et al. 2000; Zarate et al. 2006; Niciu et al. 2014; Singh et al. 2016). This might be an indication of why the sustained effects of ketamine are dependent on an intact 5-HT system (Gigliucci et al. 2013; Pham et al. 2017). Moreover, the increased FA in vHPC observed 7 days after ketamine administration could be associated to the necessary stimulation of the vHPC-IL pathway to support the antidepressant-like effects of the drug (Carreno et al. 2019). The increase of FA in the vHPC-IL network, which could be associated with positive emotions (Krystal et al. 2019), and in the CC, which could be suggestive of an increase in the activation of inter-prefrontal areas (Riva-Posse et al. 2014), favor the superior antidepressant effects of ketamine in the clinic. In fact, this increased structural changes in the vHPC-IL network also corresponds to the ketamine-induced increase in glucose consumption (Carlson et al. 2013; Lally et al. 2015; Li et al. 2016). If our observations of changes in DWI scalars are translatable to human condition, we would postulate that increased FA in the IL and DRN may signal response to antidepressant treatment. Although the present data suggest that this is a necessary condition, further research is needed to demonstrate that it is also sufficient. Most importantly, these effects of ketamine can be regarded as truly contributing to its antidepressant features, inasmuch as dissociative and psychotomimetic effects were no longer present at the time when the scan was performed, i.e. 24 h after drug administration. Ro 25-6981 produced similar increases of FA in DRN and IL, which most likely contribute to its antidepressant-like effects. Previous work has shown that the stimulation of the IL cortex projection to the DRN also elicits similar effects (Covington et al. 2010; Warden et al. 2012; Challis et al. 2014), resembling those of intra-IL or systemic ketamine (Fuchikami et al. 2015). Contrary to the results with ketamine, the Ro 25-6981-induced increase in FA in DRN and IL cortex occurred with concomitant increases in RD. Because Ro 25-6981 did not modify MBP, but increased NF200 immunostaining in gray matter areas, such as IL and DRN (see below), it is possible that the increase in RD in those areas may reflect increases in the number of crossing fibers and/or dendritic processes (Choi et al. 2015; Winklewski et al. 2018).

Overall, our results are also coincident with previous work showing that a prefrontal-subcortical activation after ketamine treatment, which resulted from a transient activation of glutamate signaling in the mPFC, is crucial for a rapid antidepressant effect (Gerhard et al. 2016; Aleksandrova et al. 2017). Indeed, both ketamine and Ro 25-6981 infused in the mPFC produced antidepressant-like effects (Kiselycznyk et al. 2015; López-Gil et al. 2019). However, the more robust antidepressant action of ketamine compared to Ro 25-6981 could be attributed to the fact that ketamine increases prefrontal glutamate whereas Ro 25-6981 does not (Krystal et al. 2013; Jiménez-Sánchez et al. 2014; Duman et al. 2016). Ketamine also induced positive changes in structural connectomics with respect to Ro 25-6981, which might be related to its better therapeutic profile. For instance, our results show that ketamine possesses a greater nodal efficiency along time with respect to Ro 25-6981 in the NAcc, mSTR and OFC of the left hemisphere and in the vHPC of the right hemisphere. This may be associated with the reported large metabolic increase in these regions (Lally et al. 2014; Nugent et al. 2014) and, taken together, represent a better activation of reward circuitry (Zhang et al. 2013; Ionescu et al. 2018) and decreased anhedonia (Lally et al. 2014, 2015) induced by ketamine. In addition, the increased local connectivity parameters in the medial striatum after ketamine may be suggestive of enhanced response to positive stimuli (Murrough et al. 2015). Of note, these changes were observed only 1 week after drug treatment. Interestingly, ketamine also produced higher nodal clustering coefficient than Ro 25-6981 in the striatum predominantly 7 days after drug administration, which may represent an improved intrinsic connectivity after ketamine administration and suggests that topological modifications of specific nodes are required for the sustained antidepressant effects of ketamine.

Because FA is highly sensitive to microstructural changes, but less specific to the type of change, we set out to examine whether MBP and/or NF200 were involved in the changes observed in the IL and the DRN after ketamine and Ro 25-6981. In fact, MBP expression is positively correlated with concomitant increases in FA and decreases in RD (Provenzale et al. 2015). Increases in MBP may indicate myelination of already existing or newly formed axons whereas increases in NF200 are suggestive of increased cytoskeleton neurofilaments (see below). In cortical structures, NF200 is used as marker of pyramidal cells (Saito et al. 2014). Our results showed that both ketamine and Ro 25-6981 increased NF200 immuno-labeling in the deep layers of the IL and in the DRN. Most importantly, the increase in NF200 was observed only in the deep (5 and 6) layers of the mPFC where pyramidal neurons that project to subcortical areas (including the DRN) are located (Gabbott et al. 2005; Fuchikami et al. 2015). MBP also exhibited an increase in the deep layers of IL after only 24 h, as observed in mice chronically treated with the classical antidepressant venlafaxine in parallel to its antidepressant-like effects (Zhang et al. 2019). Neurofilament staining provides a global measure of fiber integrity at subcellular resolution, which allows the visualization of subtle changes in fiber organization that are predicted by DWI analyses. The precise function of neuro-filaments remains poorly understood, but increased NF200 implies increased axonal density that should favor high-velocity nerve conduction (Barry et al. 2012). The light-chain constitutes the backbone of neurofilaments whereas the heavy-chain (NF200) is involved in the formation of side arms (Gordon 2020). Thus, NF200 might regulate the formation of new spines (Yuan et al. 2015), which could contribute to ketamine-induced increase in spine density in mPFC (Li et al. 2010; Fuchikami et al. 2015). In one study, the same dose of Ro 25-6981 did not change dendritic spine number in the cortex, but the measure was carried out only 30 min after Ro 29-6981 administration (Gupta et al. 2015), which might have not been sufficient for such changes to occur.

Although neither MRI nor NF200 and MBP immunostaining can distinguish ascending from descending fibers, our results suggest that both ketamine and Ro 25-6981 stimulate the projection from IL to DRN, an assumption that is based on several premises: first, the increases in FA and NF200 are confined to the IL; second, the deep layers of IL are those that project to subcortical structures, including the DRN (Gabbott et al. 2005; Fuchikami et al. 2015); third, if both drugs had an impact on ascending serotonergic fibers, similar changes would be also expected to occur in superficial layers of the IL (Blue et al. 1988; Belmer et al. 2019). Ketamine-induced increases of FA measures as well as neurofilament and myelin immunolabeling in IL and DRN areas would imply that subtle deficits in neurofilaments and myelin in these structures would lead to impaired neuronal communication that might be involved in some forms of depression. In this regard, previous studies have revealed significant reductions in FA (Nugent et al. 2019), NF200 (Law and Harrison 2003) and myelination (Sacchet and Gotlib 2017) in corticolimbic structures in depression. In addition, reduced myelination in the mPFC has been found in the chronic social defeat model (Lehmann et al. 2017).

### Limitations of the study

The first obvious limitation relates to the small number of animals per treatment and the need for further research to confirm our results. A second limitation is the lack of a control group to account for changes in FA over time. However, while this may be a true concern for rats under development, our rats were 10–12 weeks old and can be considered as adults so that little change in FA is expected in our 2-week experiment. A further limitation to the present study is the absence of a comparator group treated, for instance, with an acute dose of an SSRI. It would be expected that a single dose of a SSRI would not cause any change in diffusion metrics. In support of this view it has been shown that a sustained administration of fluoxetine is needed to increase average FA (Delorenzo et al. 2013) and cytoskeletal proteins (Guest et al. 2004; Reinés et al. 2008; Sanna et al. 2017). Finally, the other limitation of the study is that our results report the effects of ketamine and Ro 25-6981 in naïve animals as opposed to following stress exposure. There is evidence, however, that ketamine produces similar reductions in immobility in the FST 24 h after its administration in both naïve and chronic mild stress model of depression (Franceschelli et al. 2015), which supports the view that ketamine evokes comparable effects in stressed and unstressed animals. Further research is needed to ascertain the effects of acute ketamine or Ro 25-6981, but previous work has shown that rats exposed to a chronic stress model exhibit a depressive-like behavior and a reduced neurofilament staining in the brain and that prolonged administration of conventional antidepressant drugs is needed to revert both alterations (Sanna et al. 2017).

## Conclusion

In summary, the present study provides new evidence that specific changes in microstructural patterns of connectivity differentiate the effects of ketamine and Ro 25-6981. The antidepressant-like effects of both drugs appear to be associated with increases in NF200 and MBP. We also hypothesize that the increased FA in IL and DRN may be a good index of response to antidepressant treatment, which supports the view that characterizing initial microstructural changes in such areas with DWI may be particularly helpful for early identification of depressive states and effective response to treatment. Further, the local structural changes in connectomics could be associated to the activation of neuro-circuitry implicated in improving mood and reward.

## Supplementary Information

Below is the link to the electronic supplementary material.

Supplementary file1 (DOCX 125 KB)

## Data Availability

The datasets generated during and/or analyzed during the current study are available from the corresponding author on reasonable request.
