# Supplementary material for: Structural connectivity and subcellular changes after antidepressant doses of ketamine and Ro 25-6981 in the rat: an MRI and immuno-labeling study
Source: Brain Struct Funct. 2021 Aug 7;226(8):2603–16. doi: 10.1007/s00429-021-02354-0 (PMC8448713; doi:10.1007/s00429-021-02354-0)
Supplement: Supplementary file 1 — Supplementary file1 (DOCX 125 KB) [file 429_2021_2354_MOESM1_ESM.docx]

**Supplementary Table S1.** Effects of ketamine (25 mg/kg) on fractional anisotropy (FA), median diffusivity (MD), axial diffusivity (AD) and radial diffusivity (RD) in different regions of the rat brain

|  | **FA** | | | | **MD (x10^-3^)** | | | | **AD (x10^-3^)** | | | | **RD (x10^-3^)** | | | |
| --- | --- | --- | --- | --- | --- | --- | --- | --- | --- | --- | --- | --- | --- | --- | --- | --- |
| **Region** | PRE | 24 h | 7 days | Change | PRE | 24 h | 7 days | Change | PRE | 24 h | 7 days | Change | PRE | 24 h | 7 days | Change |
| **r-DRN** | .299 | .340*** | .327*** | **↑** | .711 | .689* | .658***^,##^ | ↓ | .920 | .975 | .975 | = | .581 | .562 | .548** | ↓ |
| **l-DRN** | .317 | .345*** | .344*** | ↑ | .696 | .675 | .654***^,#^ | ↓ | .917 | .945 | .909 | = | .588 | .554** | .536*** | ↓ |
| **r-IL** | .192 | .199 | .207* | ↑ | .688 | .697 | .689 | = | .819 | .810 | .804 | = | .638 | .623 | .611* | ↓ |
| **l-IL** | .187 | .201 | .211* | ↑ | .689 | .683 | .677 | = | .818 | .817 | .788 | = | .632 | .615 | .611* | ↓ |
| **r-PrL** | .179 | .182 | .186 | = | .670 | .674 | .671 | = | .787 | .795 | .792 | = | .609 | .602 | .597 | = |
| **l-PrL** | .179 | .179 | .189 | = | .674 | .682 | .672 | = | .799 | .803 | .801 | = | .634 | .619 | .615 | = |
| **r-ACg** | .172 | .181 | .182 | = | .720 | .710 | .705 | = | .852 | .844 | .850 | = | .660 | .651 | .636* | ↓ |
| **l-ACg** | .180 | .188 | .190 | = | .726 | .726 | .719 | = | .858 | .850 | .848 | = | .667 | .656 | .648 | = |
| **r-dHPC** | .188 | .197 | .204 | = | .709 | .696 | .689 | = | .838 | .828 | .828 | = | .649 | .631 | .620* | ↓ |
| **l-dHPC** | .186 | .190 | .202 | = | .704 | .699 | .691 | = | .839 | .833 | .836 | = | .652 | .638 | .630 | = |
| **r-vHPC** | .206 | .214 | .223* | ↑ | .703 | .686 | .692 | = | .837 | .843*** | .854***^,###^ | **↑** | .628 | .614 | .608 | = |
| **l-vHPC** | .205 | .219 | .221* | ↑ | .702 | .691 | .692 | = | .835 | .839 | .842 | = | .628 | .612 | .607 | = |
| **r-Amy** | .280 | .288 | .286* | ↑ | .649 | .638 | .639 | = | .856 | .824 | .853 | = | .575 | .545** | .542** | ↓ |
| **l-Amy** | .274 | .276 | .281 | = | .655 | .635 | .649 | = | .858 | .823 | .834 | = | .568 | .543* | .537** | ↓ |
| **r-NAcc** | .283 | .294* | .305* | ↑ | .595 | .589 | .585 | = | .782 | .779 | .782 | = | .519 | .501 | .491** | ↓ |
| **l-NAcc** | .302 | .305 | .316 | = | .607 | .599 | .597 | = | .785 | .776 | .789 | = | .513 | .502 | .501 | = |
| **r-OFC** | .195 | .204 | .206** | ↑ | .660 | .653 | .654 | = | .791 | .790 | .789 | = | .610 | .591 | .593 | = |
| **l-OFC** | .202 | .204 | .214 | = | .668 | .675 | .662 | = | .809 | .817 | .805 | = | .612 | .613 | .600 | = |
| **r-THL** | .262 | .272 | .273 | = | .654 | .633* | .640 | ↓ | .839 | .813 | .809 | = | .576 | .553* | .546** | ↓ |
| **l-THL** | .255 | .266 | .263 | = | .652 | .637 | .642 | = | .838 | .822 | .811 | = | .570 | .547* | .546* | ↓ |
| **r-STR(m)** | .239 | .239 | .239 | = | .633 | .627 | .628 | = | .809 | .789 | .803 | = | .563 | .548 | .542 | = |
| **l-STR(m)** | .237 | .233 | .236 | = | .647 | .640 | .646 | = | .821 | .802 | .814 | = | .566 | .553 | .548 | = |
| **r-STR(l)** | .223 | .229 | .230 | = | .617 | .613 | .614 | = | .780 | .766 | .781 | = | .553 | .539 | .533 | = |
| **l-STR(l)** | .224 | .222 | .225 | = | .632 | .624 | .630 | = | .796 | .778 | .789 | = | .558 | .544 | .542 | = |
| **r-CC** | .437 | .444 | .452* | ↑ | .688 | .678 | .677 | = | 1.05 | 1.04 | 1.02 | = | .518 | .506 | .492* | ↓ |
| **l-CC** | .440 | .443 | .455* | ↑ | .686 | .681 | .677 | = | 1.06 | 1.05 | 1.04 | = | .523 | .507 | .497* | ↓ |

Animals (*n* = 5) were subjected to three scans: one week before (PRE), and 24 h and 7 days after ketamine treatment. r and l denote right and left hemispheres, respectively. Arrows indicate the direction of change. Results are expressed as mean (SEM values are omitted for the sake of clarity. =, no significant change, **p* < 0.05, ***p* < 0.01, ****p* < 0.001, different from PRE values and ^#^*p* < 0.05, ^##^*p* < 0.01, ^###^*p* < 0.001, different from 24 h values (Duncan’s multiple comparisons test). Abbreviations: DRN, dorsal raphe nucleus; IL, infralimbic cortex; PrL, prelimbic cortex; ACg, anterior cingulate cortex; dHPC, dorsal hippocampus; vHPC, ventral hippocampus; Amy, amygdala; NAcc, nucleus accumbens; OFC, orbitofrontal cortex; THL, thalamus; STR(m), medial striatum; STR(l), lateral striatum and CC, corpus callosum.

**Supplementary Table S2.** Effects of Ro 25-6981 (10 mg/kg) on fractional anisotropy (FA), median diffusivity (MD), axial diffusivity (AD) and radial diffusivity (RD) in different regions of the rat brain

|  | **FA** | | | | **MD (x10^-3^)** | | | | **AD (x10^-3^)** | | | | **RD (x10^-3^)** | | | |
| --- | --- | --- | --- | --- | --- | --- | --- | --- | --- | --- | --- | --- | --- | --- | --- | --- |
| **Region** | PRE | 24 h | 7 days | Change | PRE | 24 h | 7 days | Change | PRE | 24 h | 7 days | Change | PRE | 24 h | 7 days | Change |
| **r-DRN** | .319 | .335* | .329 | **↑** | .699 | .696 | .680 | = | .924 | .960 | .918 | = | .561 | .588* | .568 | **↑** |
| **l-DRN** | .337 | .344 | .352* | **↑** | .668 | .704** | .654^###^ | **↑** | .906 | .955 | .893 | = | .525 | .562*** | .535 | **↑** |
| **r-IL** | .180 | .201* | .194 | **↑** | .695 | .707 | .726** | **↑** | .817 | .820 | .858 | = | .635 | .638 | .675***^,###^ | **↑** |
| **l-IL** | .178 | .211*** | .208*** | **↑** | .678 | .688 | .683 | = | .808 | .830 | .837 | = | .613 | .619 | .610 | = |
| **r-PrL** | .178 | .174 | .181 | = | .667 | .687 | .686 | = | .790 | .809 | .813 | = | .611 | .625 | .621 | = |
| **l-PrL** | .180 | .189 | .192 | = | .684 | .693 | .691 | = | .807 | .821 | .826 | = | .620 | .622 | .624 | = |
| **r-ACg** | .192 | .194 | .194 | = | .691 | .710 | .717* | **↑** | .823 | .841 | .848 | = | .630 | .654* | .652 | **↑** |
| **l-ACg** | .180 | .188 | .186 | = | .707 | .730* | .720 | **↑** | .840 | .861 | .848 | = | .643 | .655* | .657 | **↑** |
| **r-dHPC** | .184 | .184 | .184 | = | .695 | .710 | .711 | = | .825 | .839 | .838 | = | .631 | .647 | .650 | = |
| **l-dHPC** | .183 | .180 | .174 | = | .698 | .712 | .718* | **↑** | .827 | .841 | .847 | = | .629 | .645 | .645 | = |
| **r-vHPC** | .212 | .200 | .196 | = | .683 | .706* | .703* | **↑** | .819 | .839 | .838 | = | .606 | .631* | .630* | **↑** |
| **l-vHPC** | .215 | .201 | .197 | = | .681 | .698 | .697 | = | .824 | .838 | .833 | = | .605 | .627* | .625 | **↑** |
| **r-Amy** | .273 | .267 | .255* | ↓ | .640 | .678*** | .668** | **↑** | .822 | .868 | .836 | = | .547 | .582** | .578** | **↑** |
| **l-Amy** | .278 | .270 | .258* | ↓ | .641 | .669* | .658 | **↑** | .828 | .858 | .839 | = | .545 | .566 | .570* | **↑** |
| **r-NAcc** | .293 | .274 | .276 | = | .577 | .601* | .600* | **↑** | .744 | .764 | .767 | = | .496 | .521* | .525** | **↑** |
| **l-NAcc** | .293 | .281 | .283 | = | .580 | .611** | .605* | **↑** | .745 | .779 | .774 | = | .503 | .533** | .525* | **↑** |
| **r-OFC** | .221 | .209 | .201* | ↓ | .650 | .677* | .691*** | **↑** | .796 | .823 | .834 | = | .577 | .604* | .620*** | **↑** |
| **l-OFC** | .218 | .206 | .203* | ↓ | .658 | .675 | .674 | = | .792 | .806 | .801 | = | .586 | .608* | .600 | **↑** |
| **r-THL** | .280 | .275 | .274 | = | .630 | .652* | .653* | **↑** | .803 | .835 | .828 | = | .539 | .562 | .561 | = |
| **l-THL** | .275 | 272 | .274 | = | .633 | .653 | .646 | = | .810 | .833 | .828 | = | .544 | .560 | .559 | = |
| **r-STR(m)** | .230 | .229 | .232 | = | .612 | .630* | .633* | **↑** | .769 | .785 | .791 | = | .544 | .558 | .551 | = |
| **l-STR(m)** | .231 | .230 | .235 | = | .625 | .644 | .644 | = | .781 | .799 | .808 | = | .550 | .561 | .565 | = |
| **r-STR(l)** | .224 | .220 | .222 | = | .604 | .623 | .625 | = | .753 | .768 | .774 | = | .538 | .556 | .555 | = |
| **l-STR(l)** | .224 | .224 | .229 | = | .618 | .637 | .625 | = | .767 | .784 | .796 | = | .546 | .556 | .561 | = |
| **r-CC** | .464 | .454 | .457 | = | .661 | .690** | .680 | **↑** | 1.04 | 1.06 | 1.07 | = | .480 | .505* | .494 | **↑** |
| **l-CC** | .467 | .459 | .459 | = | .657 | .689** | .677 | **↑** | 1.02 | 1.02 | 1.02 | = | .476 | .504** | .493 | **↑** |

Animals (*n* = 5) were subjected to three scans: one week before (PRE), and 24 h and 7 days after ketamine treatment. r and l denote right and left hemispheres, respectively. Arrows indicate the direction of change. Results are expressed as mean (SEM values are omitted for the sake of clarity. =, no significant change, **p* < 0.05, ***p* < 0.01, ****p* < 0.001, different from PRE values and ^###^*p* < 0.001, different from 24 h values (Duncan’s multiple comparisons test). Abbreviations as in Supplementary Table S1.


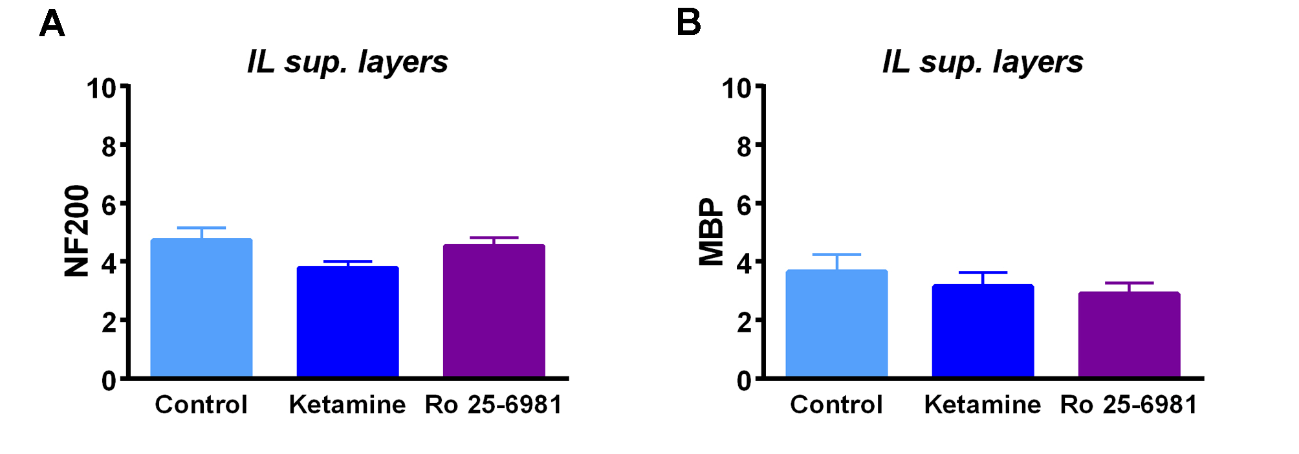


**Supplementary Figure S1.** Changes in immunolabeling for NF200 (A) and MBP (B) 24 h after the administration of vehicle (Control, 50% DMSO, 1 ml/kg, i.p.), ketamine (25 mg/kg, i.p.) or Ro 25-6981 (10 mg/kg, i.p.). Analyses were performed in superficial layers of IL. *n* = 5 animals per group.
